# Projecting introgression from domestic cats into European wildcats in the Swiss Jura

**DOI:** 10.1111/eva.12968

**Published:** 2020-05-05

**Authors:** Claudio S. Quilodrán, Beatrice Nussberger, David W. Macdonald, Juan I. Montoya‐Burgos, Mathias Currat

**Affiliations:** ^1^ Department of Zoology University of Oxford Oxford UK; ^2^ Department of Genetics and Evolution ‐ Anthropology Unit Laboratory of Anthropology Genetics and Peopling History University of Geneva Geneva Switzerland; ^3^ Institute of Evolutionary Biology and Environmental Studies University of Zurich Zurich Switzerland; ^4^ Department of Zoology Wildlife Conservation Research Unit The Recanati‐Kaplan Centre University of Oxford Oxford UK; ^5^ Department of Genetics and Evolution Laboratory of Vertebrate Evolution University of Geneva Geneva Switzerland; ^6^ Institute of Genetics and Genomics in Geneva (IGE3) Geneva Switzerland

**Keywords:** admixture, approximate Bayesian computation, hybridization, interbreeding, invasive species, spatially explicit simulations

## Abstract

Hybridization between wild and domesticated organisms is a worldwide conservation issue. In the Jura Mountains, threatened European wildcats (*Felis silvestris*) have been demographically spreading for approximately the last 50 years, but this recovery is coupled with hybridization with domestic cats (*Felis catus*). Here, we project the pattern of future introgression using different spatially explicit scenarios to model the interactions between the two species, including competition and different population sizes. We project the fast introgression of domestic cat genes into the wildcat population under all scenarios if hybridization is not severely restricted. If the current hybridization rate and population sizes remain unchanged, we expect the loss of genetic distinctiveness between wild and domestic cats at neutral nuclear, mitochondrial and Y chromosome markers in one hundred years. However, scenarios involving a competitive advantage for wildcats and a future increase in the wildcat population size project a slower increase in introgression. We recommend that future studies assess the fitness of these hybrids and better characterize their ecological niche and their ecological interactions with parental species to elucidate effective conservation measures.

## 1 INTRODUCTION

Interbreeding between evolutionarily significant units leading to hybridization is of growing concern in evolutionary and conservation biology. Newly developed molecular tools have revealed the influence of hybridization on the evolution of many taxa, and this has led to concern about the impact of human‐induced hybridization on biodiversity (McFarlane & Pemberton, 2019).

Hybridization may result in increases in genetic diversity and opportunities for adaptation preceding changing environmental conditions (Taylor, Larson, & Harrison, 2015). However, it may also reduce fitness, for example, due to outbreeding depression (e.g. Muhlfeld et al., 2009), the introduction of maladaptive genes (Kidd, Bowman, Lesbarreres, & Schulte‐Hostedde, 2009), or competition with hybrids (e.g. Ryan, Johnson, & Fitzpatrick, 2009). This is particularly important when it concerns domesticated animals, which have been artificially selected according to human needs and lifestyles, and the spread of their genes in wild populations may have serious consequences for the fitness, ecology and behaviour of wild animals (Driscoll, Macdonald, & O'Brien, 2009). 

Historically, European wildcats (*Felis silvestris silvestris* Schreber, 1778) were widely distributed in Europe (Sommer & Benecke, 2006), but they were brought close to extinction in a number of countries due to habitat loss and persecution during the 19th and 20th centuries (Stahl & Artois, 1994). In Switzerland, they were considered virtually extinct, with no firm scientific evidence of their presence for 25 years from 1943 to 1968 (Nussberger, Weber, Hefti‐Gautschi, & Lüps, 2007). A change in the Swiss federal hunting law protected this species beginning in 1962 (Duelli & Agosti, 1994). Since that time, wildcat sightings have increased over the last 50 years (Dötterer & Bernhart, 1996; Nussberger, Wandeler, Weber, & Keller, 2014). The demographic recovery of the European wildcat in Switzerland appears to be the result of successful species protection (e.g. Say, Devillard, Léger, Pontier, & Ruette, 2012). However, this recent demographic expansion has occurred in an area that was already colonized by non‐indigenous domestic cats (*Felis catus*) (Nussberger, Currat, Quilodran, Ponta, & Keller, 2018).

Domestic cats were first introduced to Europe by the Romans (Faure & Kitchener, 2009), but they are now distributed worldwide due to their association with humans as pets. Domestic cats originated from a distinct wildcat, *Felis lybica*, which was domesticated approximately 9,500 years ago in the Near East (Driscoll et al., 2007). This domestication process altered the cats’ rate of reproduction, resistance to disease, and behaviour (Oliveira, Godinho, Randi, & Alves, 2008) and produced morphological changes in size and colour (Daniels et al., 2001). Charles Darwin observed their morphological differences when he compared them to wildcats. For example, he noted a longer intestine, a change that is primarily attributed to the introduction of kitchen scraps including vegetal ingredients in the diet of domestic cats, which differs from the strictly carnivorous diet of their wild ancestors (Darwin, 1868). Another important change is that domestic cats are social and tolerant to humans, whereas wildcats are territorial and solitary (Driscoll et al., 2009). Domestic cats also carry genotypes that differ from those of all other subspecies of wildcats (Driscoll et al., 2009). Even if the taxonomic status of European wildcats and domestic cats is still controversially discussed (Kitchener et al., 2017) and independently of the degree of genomic divergence of both taxa, here, we consider both cat taxa as two distinct evolutionarily significant units, especially since domestic cats have—at least to some extent—a human‐biased evolution.

The wildcat genome is introgressed by domestic cat genes at a higher frequency than the domestic cat genome is introgressed by wildcat genes (Table 1, Nussberger et al., 2014). We previously showed that these asymmetric introgression levels are best explained by the range expansion of wildcats according to spatially explicit simulations that take into account ecological and genetic characteristics (Nussberger et al., 2018). This observation is in accordance with the general expectation of neutral genetic markers in organisms experiencing range expansion and hybridizing with closely related taxa that are already present in the area (Currat, Ruedi, Petit, & Excoffier, 2008). In the case of the wildcats of the Swiss Jura, their recolonization started in an area already occupied by domestic cats. The demographic imbalance at the wave front of their range expansion resulted in asymmetric introgression between the two types of cats.

**Table 1:** Observed introgression and parameter values used to simulate interbreeding between European wildcats and domestic cats (modified from Quilodrán et al. (2019) and Nussberger et al. (2018))

	Wildcat	Domestic cat
Genetic introgression		
Autosomal	12.5%	2.15%
mtDNA	15.6%	1.08%
Y chromosome	4.82%	0%
Model parameters		
Generation time (years)	3	3
Interbreeding success rate (γ)	0–0.4	0–0.4
Growth rate (*r*)	1.0	1.0
Migration rate (*m*)	0.18	0.18
Carrying capacity (*K*)	12	70

Building on Nussberger et al. (2018), we further improved the accuracy of this ecological and genetic model in explaining the current introgression between the cats, by integrating the solitary behaviour of wildcats during range expansion (Quilodrán, Nussberger, Montoya‐Burgos, & Currat, 2019). The solitary behaviour of wildcats is supported by the ecological knowledge of the species (Corbett, 1979). A model of solitary dispersal improves the accuracy of simulations by 10 to 30% compared to random or gregarious dispersal, depending on the genetic markers that are used (Quilodrán et al., 2019). Those previous simulation studies were aimed at understanding how wildcats have recolonized their habitat during the past 50 years. Our main aim in the current study is to project possible future amounts of introgression under different ecological conditions, including varying competition and population sizes, in order to explore the implications in terms of conservation of wildcats in the Swiss Jura. To achieve this objective, we characterize the interbreeding rate that best explains current patterns of introgression at nuclear, mitochondrial and Y chromosome markers by refining the previously developed genetic and demographic models. Though the previous simulation studies included competition within populations, they ignored the competition between cat populations (Nussberger et al., 2018; Quilodrán et al., 2019). However, competition for environmental resources has been suggested to be important, with wildcats potentially presenting an advantage after antagonistic encounters (Gil‐Sánchez, Jaramillo, & Barea‐Azcón, 2015). Here, we thus include competition between the two types of cats. We also use an approximate Bayesian computation method to better estimate the value of interbreeding, instead of the non‐linear regression method used in the previous approach (Nussberger et al., 2018; Quilodrán et al., 2019).

## 2 METHODS

### 2.1 Simulations

To explain the current level of introgression and project future scenarios of hybridization between cats, we performed a series of ecological and genetic simulations. The level of introgression is considered to be represented by the proportion of genes from either recombinant (autosomal markers) or non‐recombinant genomic sections (mtDNA and Y chromosome markers) that are present in a population *i* at time *t* but sampled in population *j* at time *t* + 1. We simulated neutral genetic diversity in a spatially explicit framework by using a modified version of the software SPLATCHE3 (Currat, Arenas, Quilodrán, Excoffier, & Ray, 2019). We modified the migration model from the original version as described below. This software performed the simulations in two main steps: (a) forward simulations depending on the demographic parameters and (b) backward coalescent simulations conditioned by the first step, to estimate the proportion of introgression between interacting taxa (Currat et al., 2008).

The area of the distribution of a given taxon is represented by a grid of demes organized as stepping‐stones (Kimura & Weiss, 1964). A second taxon is represented with a superimposed grid of demes. The gene flow between neighbouring demes belonging to the same taxon is controlled by the parameter *m* (migration rate), while the gene flow between different taxa is regulated by the parameter *γ* (interbreeding success rate). The value of *m* corresponds to the proportion of individuals in each deme emigrating towards neighbouring demes at each generation, while *γ* is the probability that an encounter between the two parental taxa will result in viable, fertile offspring. We used the hybridization model implemented in SPLATCHE3. When *γ* = 0, there is no interbreeding between taxa, and when *γ* = 1, reproduction is panmictic. Any intermediate value indicates that mating is not random between interacting taxa.

The population density of each deme is logistically regulated by using the parameters *r* (growth rate) and *K* (carrying capacity). *N* and *K* correspond to effective population sizes. Interspecific competition may occur according to the Lotka–Volterra model, in which case the coefficient of the competition exerted by taxa *i* on taxa *j* (*α_ij_*)*_,_* takes values between 0 and 1. A value of *α_ij_* = 0 means that there is no competition between individuals *i* and *j*, whereas a value of 1 indicates that individuals *i* exert the same amount of competition as the individuals *j* on taxa *j* (Lotka, 1932; Volterra, 1928).

We used a modified dispersion model that was not implemented in the original version of SPLATCHE3 but was described in Quilodrán et al. (2019). To better represent territorial or solitary behaviour, this model considers the direction of dispersal to be negatively correlated with the density in neighbouring demes. The probability that each migrant from a focal deme may enter deme *i* is computed as $1/N_{i}/\sum_{j=1}^{N}\left(1/N_{j}\right)$ , where *N* is the population size of a deme *i* or *j*, while *n* represents all neighbour demes potentially colonized from the focal deme. This model was shown to explain current levels of introgression in wildcats and domestic cats better than the original random dispersal model implemented in SPLATCHE3 or a model in which dispersal is higher in already colonized areas (Quilodrán et al., 2019). We therefore use this negative density‐dependent model of spatial dispersal to project future scenarios of introgression between cats.

### 2.2 Implementation on wildcats

To assess the expected levels of the introgression of domestic genes into wildcats, we performed spatially explicit simulations of the well‐documented case of wildcat demographic recovery in the Swiss Jura Mountains (Nussberger, Greminger, Grossen, Keller, & Wandeler, 2013; Nussberger et al., 2007, 2014). We used demographic parameter values and observed introgression based on previously published studies simulating the hybridization between the two types of cats (Nussberger et al., 2018; Quilodrán et al., 2019), as shown in Table 1.

The genetic data set used to estimate introgression levels consisted of data from 68 autosomal SNP markers, four mtDNA SNP markers and two Y chromosome SNP markers. All of these markers are considered to be neutral and are highly differentiated between wild and domestic cats (Nussberger et al., 2013). The introgression level represents the proportion of individuals in a population that carry introgressed genes from the other population (Nussberger et al., 2014).

**Figure 1:**
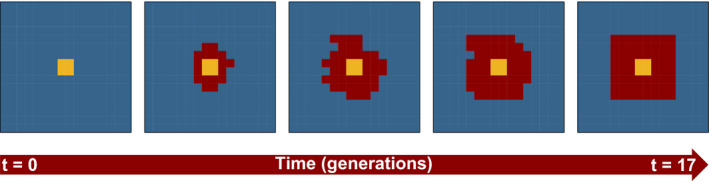
Simulation of hybridization between the European wildcat and the domestic cat in a virtual area representing the Swiss Jura. The red colour represents the hybrid zone, while the yellow and blue colours denote wildcat and domestic cat ranges (modified from Nussberger et al. (2018))

We used an array of 256 demes of 25 km^2^ (~6,400 km^2^) to roughly characterize the Swiss Jura Mountains (Figure 1). This array represents a habitat that is exclusively available for wildcats (16 demes) in the core area, a habitat suitable for both wild and domestic cats (48 demes) in the surroundings of the core, and a habitat exclusively used by domestic cats (192 demes) at the periphery. At the beginning of the simulations, the wildcats start to colonize an area that is already occupied by domestic cats (blue colour in Figure 1) and spatially expand their distribution range over 50 years (*t* = 17, considering three years per cat generation, red colour in Figure 1). This represents the historical condition of hybridization between cats in the Swiss Jura. We explored various values of the interbreeding success rate (*γ*) to identify the one that best explains the current levels of introgression in the two types of cats (see below).

To project future introgression levels in the two species, we added more generations to our most likely combination of parameters estimated from historical information. We calculate the resulting values for 100 years from now and for the number of generations needed to achieve an introgression rate of 50%, a value at which it would be difficult to differentiate between the two types of cats. Note that these expectations are valid only for neutral loci and are based on the assumption that current parameters are constant over time.

While previous simulations included competition within populations (*α_ii_*), they ignored the competition between cat populations (*α_ij_*) (Nussberger et al., 2018; Quilodrán et al., 2019), which is suggested to be important, with wildcats presenting an advantage over domestic cats (Gil‐Sánchez et al., 2015). Hereafter, competition between populations for the shared available resources (e.g. territory, food) is either considered to be absent between the cats (*α*_wild‐domestic_ = *α*_domestic‐wild_ = 0) or to occur with a competitive advantage for wildcats (*α*_wild‐domestic_ = 1 and *α*_domestic‐wild_ = 0). A competitive advantage for wildcats means that they may exclude domestic cats in areas where they meet. These models are referred to as the “no competition” and “competition” models, respectively. A range of competition values (0 ≤ α_wild‐domestic_ ≥ 1) is also analysed in Appendix S1. Note that competition within populations, both domestic and wild, is always included in our simulations using logistic demographic regulation.

### 2.3 Approximate Bayesian computation for model choice

We used the approximate Bayesian computation approach (ABC, Beaumont, Zhang, & Balding, 2002) to discriminate the best model with or without competition and to estimate the value of the interbreeding success rate (*γ*) that best explained the observed proportions of introgression in the two types of cats, which were previously estimated from SNP markers (Nussberger et al., 2014). This procedure compares the resulting proportion of introgression in the two types of cats, associated with a range of *γ* values simulated with SPLATCHE3, with the observed proportion of introgression estimated from empirical data. Previous studies have estimated that *γ* values range between 4% and 9% on the basis of considering a non‐linear regression method instead of an ABC approach and have not included the effect of competition between the two types of cats (Nussberger et al., 2018; Quilodrán et al., 2019). We obtained the average posterior probability for each model of competition and computed the Bayes factor. This factor reveals the support for a given model relative to another candidate model (Csilléry, Blum, Gaggiotti, & François, 2010). A Bayes factor in the range of 1–3.2 is considered weak, while 3.2–10 is considered substantial, and 10 or higher is considered strong (Kass & Raftery, 1995). We also estimated the goodness of fit (GOF) of each model to our observed data set. This was performed with the prior distributions. The *p*‐value associated with the GOF test evaluates whether significantly different summary statistics are obtained with the simulated models compared to those based on empirical observations. If this *p*‐value is lower than 5%, then the model is considered to fit the observations poorly. The 95% confidence interval of the *γ* values best explaining the summary statistics was also computed. We performed these analyses for each genetic marker and for all observed genetic markers taken together. We used neural networks to assess the probabilities of the models and parameter estimates with a 5% tolerance level. The tolerance level is the proportion of retained simulations that result in the simulated introgression values closest to the observed introgression values. In this case, we retained the best 1,605 simulations (5%) out of the 32,100 simulations available for each competition model and genetic marker. We also tested the resulting values at tolerance levels of 10% and 15% (Table S2). The ABC analyses were performed with the *R* package “*abc*” 2.1 (Csilléry, François, & Blum, 2012).

### 2.4 Projection of future level of introgression

Once the parameter values that best explained the current level of introgression were estimated via the ABC approach, we projected four different scenarios further in time: (a) “No change,” in which all parameters that explain the current level of introgression remain the same (*N*_wild_ < *N*_dom_); (b) “Equal size” (*N*_wild_ = *N*_dom_), in which the population size of wildcats increases in each deme until it becomes equal to the population size of domestic cats in the countryside (determined by the carrying capacity, i.e. *K*_wild_ = *K*_dom_); (c) “Larger size” (*N*_wild_ > *N*_dom_), in which the population size of wildcats increases until it doubles the population size of domestic cats in the countryside (*K*_wild_ = 2*K*_dom_); (d) “Stop interbreeding” (*N*_wild_ < *N*_dom_), in which the habitat that is available exclusively to wildcats continues to expand throughout the shared demes, and the reproductive encounters between the two types of cats that lead to hybridization therefore stop (*γ* = 0).

## 3 RESULTS

### 3.1 Models of competition

The two models—with (*α*_wild‐domestic_ = 1) and without competition (*α*_wild‐domestic_ = 0)—present a similar goodness of fit (*p*‐values > .05, Table 2). This means that both models are able to reproduce the observed levels of introgression in the two types of cats. However, the model without competition between the cats is the model that generally explains at best the current level of introgression (see Table 2). The two models are equally likely for autosomal markers, but considering all markers together, the model without competition fits better (Bayes factor: 36 times as likely as the competition model, which provides strong support in favour of this model). The 5% level of tolerance used for the retention of better simulations is not high enough for model selection and the estimation of *γ* values for the mtDNA and Y chromosome markers using the competition model. By increasing this tolerance to 10% and 15%, the Bayes factor distinguishes a better fit for the model without competition (see Table S2). The results for the autosomal markers and all markers taken together are consistent at any level of tolerance, with no competition preferred to competition.

**Table 2:** Approximate Bayesian computation (ABC) was used for model comparison and the estimation of *γ* values (mode, mean and confidence interval at 95%)

Marker	Model	Posterior probability	Bayes factor	GOF *p*‐value	*γ* mode	*γ* mean	*γ* CI 95%
Autosomal	No competition	0.58	1	.18	4.58	4.41	3.03–6.5
	Competition	0.42	1.41	.16	6.07	6.08	4.84–7.45
mtDNA	No competition	–	–	.39	7.52	9.98	6.13–17.3
	Competition	–	–	.36	–	–	–
Ychrom	No competition	–	–	.23	5.05	3.58	0–8.87
	Competition	–	–	.18	–	–	–
All	No competition	0.97	1	.22	6.01	6.29	5.18–7.59
	Competition	0.03	36.4	.19	6.11	6	4.31–7.88

Note

Five per cent of the best simulations were retained and used for model comparison and the estimation of *γ* parameter value. The Bayes factor is presented as the probability of the “no competition” model (numerator) relative to the probability of each other model (denominator). The *p*‐value of the goodness of the fit of each model (GOF) is also presented. We performed 32,100 simulations for each model of competition and genetic marker.

While a model of full competition between cats (α_wild‐domestic_ = 1) performed poorly in contrast to a model without competition (α_wild‐domestic_ = 0), competition coefficients giving an advantage for wildcats over domestic cats (0 < α_wild‐domestic_ ≤ 0.8) result in highly similar levels of introgression (Figure S1). For this reason, it is possible, if not likely, that competition has occurred during the recent range expansion of wildcats (Table S1), and it may potentially play a role in the future when the spatial dynamics of wildcats reach demographic equilibrium. Thus, in the next sections, we project future introgression under extreme values of competition (i.e. absence or full competition) to explore the range of possible outcomes that may be expected.

### 3.2 Interbreeding success rate

**Figure 2:**
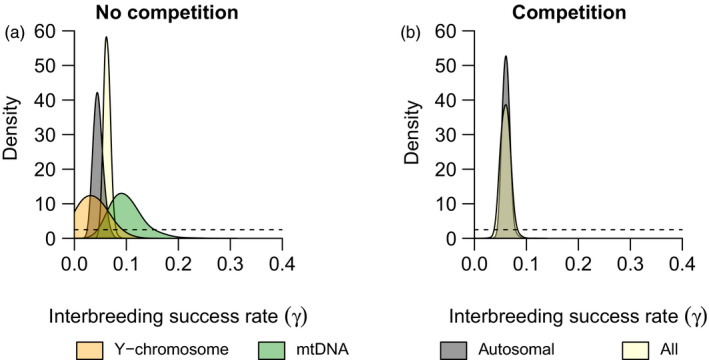
Bayesian estimation of the interbreeding success rate (*γ*) explaining the currently observed introgression between European wildcats and domestic cats. The dotted and solid lines represent the prior and posterior distributions, respectively. The estimates consider either no competition between cat populations (a) or competition with a competitive advantage of wildcats (b). Competition within populations is always included in our simulations

Considering all genetic markers and models of competition, the interbreeding success rate (*γ*) that best explains the level of introgression is estimated to be approximately 6% (Figure 2). This estimation indeed overlaps for the autosomal markers and all markers taken together in both models of competition at the 95% confidence interval (Figure 2, Table 2).

We next used *γ* values of the model without competition to project future values of introgression (i.e. mode of 4.58% for autosomal, 7.52% for mtDNA and 5.05% for Y chromosome markers, see Figure 2). We make this choice because the model without competition is best supported by the ABC approach and because the *γ* values estimated with both competition models are not significantly different in the 95% interval (Table 2).

### 3.3 Projection of future level of introgression

#### 3.3.1 “No change” scenario (*N*_wild_ < *N*_dom_)

**Figure 3:**
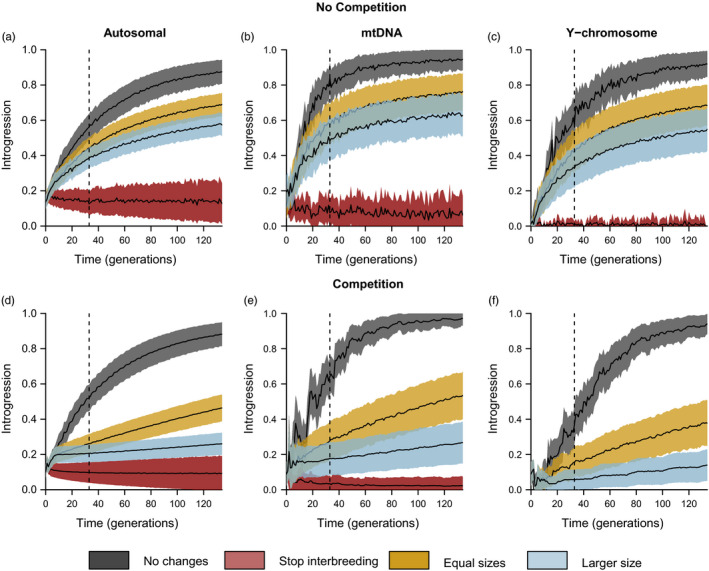
Projected introgression levels over time in European wildcats (mean ± *SD*). The vertical dotted lines denote the projection 100 years from now. The figures at the top project introgression in the absence of competition between cats, while the figures at the bottom include competition between them. Competition within populations of cats is always included in the simulations (see methods). Values are averaged over 10,000 simulations

Under the scenario of “No change,” we assume the continuation of the current level of interbreeding and population sizes (i.e. *N*_wild_ < *N*_dom_). The main expectation is that wildcats will be progressively introgressed by domestic cats at an increasing rate until becoming difficult to distinguish. Under the no competition model, 50% of the wildcat genetic pool is projected to be composed of genes from domestic cats in the 26th, 15th and 20th generations from now for the autosomal, mtDNA and Y chromosome markers, respectively (Figure 3a–c, grey areas). Following this scenario, wildcats will be assimilated into domestic cats in less than 250 years. One hundred years from now, our simulations project that 57% of the autosomal genes of wildcats will have been introgressed from domestic cats as well as 78% of their mtDNA and 68% of their Y chromosome (Figure 3a–c, dotted lines).

Similar results are obtained using the competition model, but it takes more generations to reach equal values of introgression. A level of introgression of 50% of the wildcat gene pool is obtained in the 30th generation, or 90 years from now, for the autosomal markers (Figure 3d), while this level is expected to occur in the 20th generation for mtDNA (Figure 3e) and 44th generation for the Y chromosome (Figure 3f). One hundred years from now, our simulations reveal that 53% of autosomal genes in wildcats will have been introgressed from domestic cats as well as 66% of their mtDNA and 43% of their Y chromosome (Figure 3d–f, dotted lines).

We also show that domestic cats will be much less introgressed because they demographically outnumber wildcats so overwhelmingly (Figure S2). Nevertheless, our simulations suggest that even when wildcats are massively introgressed by domestic genes under our most extreme scenario (“No change”), there will still be a small proportion of wildcat genes remaining in the gene pool of domestic cats. The proportion of wildcat introgression in domestic cats is highest in autosomal markers at 10 ± 5% (mean ± *SD*) according to the no‐competition model and 8 ± 4% according to the competition model. Among mtDNA and Y chromosome markers, the projection of future introgression is negligible in domestic cats (Figure S2).

#### 3.3.2 “Equal sizes” scenario (*N*_wild_ = *N*_dom_)

In this scenario, in which the population size of wildcats increases to a size equal to that of domestic cats, 50% introgression is reached in 46, 21 and 45 generations from now for the autosomal, mtDNA and Y chromosome markers, respectively (Figure 3a–c, yellow areas). One hundred years from now, 45% of autosomal genes in wildcats are projected to have been introgressed from domestic cats as well as 58% of their mtDNA and 43% of their Y chromosome (Figure 3a–c, dotted lines). When a competitive advantage of wildcats is included in this scenario, 26% of autosomal genes in wildcats will have been introgressed from domestic cats as well as 29% of their mtDNA and 17% of their Y chromosome in one hundred years (Figure 3d–f, dotted lines). In the model including competition, the 50% introgression level is reached after 115 generations for mtDNA markers, while this level is never reached for the autosomal markers or the Y chromosome during the 130 generations simulated further in time (Figure 3d–f, yellow areas).

Compared to the “No change” scenario, the highest level of introgression observed is approximately 20% lower for the autosomal and mtDNA markers and approximately 26% lower for the Y chromosome when considering equal population sizes and no competition between cat species (Figure 3a–c, yellow areas compared to grey areas). When competition is included, the projected introgression levels drop even further, by approximately 45% for the autosomal and mtDNA markers and approximately 60% for the Y chromosome (Figure 3d–f, yellow areas compared to grey areas).

#### 3.3.3 “Larger size” scenario (*N*_wild_ > *N*_dom_)

Introgression is further reduced when considering a population of wildcats that is double the size of the domestic cat population in the area where they coexist. With no competition, 50% introgression is reached in 76, 36 and 97 generations from now for the autosomal, mtDNA and Y chromosome markers, respectively (Figure 3a–c, sky blue areas). One hundred years from now, 39% of autosomal genes in wildcats will have been introgressed from domestic cats as well as 50% of their mtDNA and 35% of their Y chromosome (Figure 3a–c, dotted lines). When including competition, 21% of autosomal genes in wildcats will have been introgressed from domestic cats as well as 18% of their mtDNA and 6% of their Y chromosome one hundred years from now (Figure 3d–f, dotted lines).

In this scenario, even if the rate of the introgression of domestic cats into European wildcats is expected to be higher than today in the near future, it will not increase to the same extent projected under the other scenarios in the long term. A 50% introgression rate is not reached for any simulated genetic marker.

Compared to the “No change” scenario, the reduction of the level of introgression from the most extreme values is approximately 35% for the autosomal and mtDNA markers and by approximately 40% for the Y chromosome when considering a population of wildcats that is twice as large as the population of domestic cats and no competition between cat species (Figure 3a–c, sky blue areas compared to grey areas). When considering competition, the level of introgression is much lower. It decreases approximately 70% for the autosomal and mtDNA markers and 85% for the Y chromosome (Figure 3d–f, sky blue areas compared to grey areas).

#### 3.3.4 “Stop interbreeding” scenario (*N*_wild_ < *N*_dom_)

The scenario with no more interbreeding, with or without competition, is the only one in which introgression is stabilizes or is even lower than today in the near future (Figure 3, red areas). One hundred years from now, 13% of autosomal genes in wildcats will have been introgressed from domestic cats as well as 8% of their mtDNA and 1% of their Y chromosome (Figure 3a–c, dotted lines). In the model considering competition between cats, one hundred years from now, 10% of autosomal genes in wildcats are predicted to be introgressed from domestic cats as well as 3% of their mtDNA and 0% of their Y chromosome (Figure 3d–f, dotted lines). Introgression does not reach 50% for any genetic marker or model of competition. Compared to the “No change” scenario, extreme introgression values are reduced by 85% to 100% under this scenario. Unsurprisingly, the prevention of further hybridization—ideally combined with increasing wildcat numbers—is the best way to avoid massive introgression from domestic cats in the European wildcat population.

## 4 DISCUSSION

Building on previous studies that aimed to explain the current level of introgression between wildcats and domestic cats in the Swiss Jura (Nussberger et al., 2018; Quilodrán et al., 2019), we use spatially explicit simulation to show here that the amount of introgression in wildcats is set to increase in the near future unless hybridization is drastically stopped. The greatest introgression is projected under the scenarios in which the demographic parameters are no different from those of today (“No change” scenario, *N*_wild_ < *N*_dom_). Even in the cases that are the most favourable to wildcats, including a competitive advantage for wildcats and a wildcat population size equal to (*N*_wild_ = *N*_dom_) or twice that of domestic cats (*N*_wild_ > *N*_dom_), the amount of introgression still increases over time but to a lesser extent. The only exception to this increase of introgression is the “Stop Interbreeding” scenario, in which introgression rates remain similar to or smaller than the present level, even without changes to the current population sizes (*N*_wild_ < *N*_dom_). This output of massive introgression in species experiencing a range expansion is a general expectation for neutral markers, which is described in detail by Currat et al. (2008) and has been observed in several plant and animal taxa (e.g. Duminil, Caron, Scotti, Cazal, & Petit, 2006; Oswald et al., 2019). In their study, Currat et al. (2008) used spatially explicit simulations and a literature review to demonstrate that the introgression between invasive and local species is expected to be much higher into the former, even at low levels of interbreeding. This is due to the combined effect of allelic surfing at the wave front of the invasive range expansion and demographic imbalance (Klopfstein, Currat, & Excoffier, 2006). The case of European wildcats in the Swiss Jura demonstrates that the “invasive” species of the model of Currat et al. (2008) may also represent a native species, either naturally expanding its home range or recolonizing an area previously lost due to human disturbances (Quilodrán et al., 2019).

### 4.1 Current level of introgression

Previous simulation studies have demonstrated the importance of the recent range expansion of wildcats (Nussberger et al., 2018) and negative density‐dependent dispersal (Quilodrán et al., 2019) for explaining the current level of introgression. We included here a scenario involving a competitive advantage of wildcats over domestic cats. While some level of competition cannot be excluded, a model without competition better explains the current level of introgression. This adds confidence to previous simulations of wildcats expanding in the Swiss Jura that do not include a competitive advantage to explain the current level of introgression (Nussberger et al., 2018; Quilodrán et al., 2019). With the current observed level of introgression, it is still possible to genetically differentiate wildcats from domestic cats in Switzerland, as is the case in some other parts of Europe such as in Portugal, Italy and Germany (Eckert, Suchentrunk, Markov, & Hartl, 2010; Lecis et al., 2006; Nussberger et al., 2013; Oliveira, Godinho, Randi, Ferrand, Godinho, Randi, Ferrand, & Alves, 2008), although this is becoming increasingly less possible in Scotland (Senn et al., 2019) and much higher introgression has also been found in Hungary (Beaumont et al., 2001; Lecis et al., 2006). Our simulations indicate that wildcats in the Jura might face the predicament of their conspecifics in Scotland and Hungary if hybridization is not stopped and/or their numbers are not increased.

### 4.2 Interbreeding success rate

The rate of interbreeding success that best explains the current level of introgression between wildcats and domestic cats in the Swiss Jura is estimated to be approximately 6%, i.e. ~6% of interspecific reproductive encounters result in fertile hybrid offspring. Clearly, if reproductive barriers exist, they are not complete. The interbreeding success values are similar for the autosomal and Y chromosome markers. However, a higher level of interbreeding success (8%) best explains the observed mtDNA introgression. This might be explained by the more exploratory behaviour of male wildcats during the breeding period, which results in hybrid offspring carrying mtDNA from female domestic cats (Daniels et al., 2001). The estimated values of interbreeding for each genetic marker are equivalent to those previously estimated using a regression method instead of an approximate Bayesian computation approach (Nussberger et al., 2018; Quilodrán et al., 2019). Because a higher interbreeding rate is needed to explain maternal inherited markers and these markers are the most affected by future introgression, for any management programmes that are designed, a focus on the desexing of female domestic cats should potentially be considered.

### 4.3 Projected introgression in the near future

Our simulations show that introgression between wildcats and domestic cats is likely to increase in the coming decades. While our approach explores a range of plausible options, it is still limited to neutral locus expectations. Varying selective pressures, which are not included in this approach, as well as polymorphisms that are currently neutral but may become advantageous or disadvantageous in a new environmental or genetic background, could exhibit different introgression trajectories that are difficult to predict in the near future. According to our simulations, the rate at which introgression is projected to increase for neutral genetic markers depends on how population sizes change in the future. If the rates of interbreeding and the population sizes of the two types of cats remain unchanged compared to those of today, the gene pool of wildcats is expected to be massively introgressed by domestic genes within a few hundred years. Overwhelming replacement of the wildcat gene pool with domestic cat ancestry would occur even earlier for the mtDNA and Y chromosome, which markers are evolving faster. Even when a competitive advantage is simulated for European wildcats, the model projects a trend of massive, albeit slower, introgression of domestic genes in the wildcat population.

Scenarios in which the wildcat population size increases to equal or greater than that of domestic cats in the area where they coexist and a competitive advantage of wildcats is assumed might also plausibly project the future of introgression. Indeed, it seems likely that the wildcat distribution will continue to expand and that its populations will continue to increase, as they have seemingly not yet occupied all the potentially available habitat (Dötterer & Bernhart, 1996; Nussberger et al., 2014; Weber, Roth, & Huwyler, 2010). In addition, it is reasonable to hypothesize that competition between wild and domestic cats may become important to their population dynamics in the future when their spatial densities reach equilibrium. Wildcats exhibit the solitary, intrasexual territoriality that is characteristic of small felids (Macdonald, Mosser, Mosser, & Gittleman, 2010), whereas domestic cats are more sociable (Driscoll et al., 2009; Macdonald, Yamaguchi, & Kerby, 2000). Furthermore, wildcats have had a longer time to adapt their behaviour to the wild than domestic cats, so it may be speculated that they present a competitive advantage in the natural environment (Gil‐Sánchez et al., 2015). Nevertheless, even in our most (and perhaps unrealistically) optimistic scenario, under which wildcats have a carrying capacity twice as high as that of domestic cats and exert a competitive advantage over them, our simulations suggest that introgression will still slowly increase.

The outcomes of our simulations further emphasize the obvious priority of stopping hybridization to the greatest extent possible, ideally combined with increasing wildcat numbers, to avoid increased introgression, whichever model of competition turns out to be most realistic. If hybridization is not halted and the population of wildcats does not increase, overwhelming, imminent introgression, implying the de facto extirpation of the wildcat, is almost certain.

### 4.4 Considerations about introgression consequences

Under high levels of introgression, it will be biologically, legally and ethically difficult to define the status of hybrids relative to their parental stock, both genotypically and phenotypically. The consequences of introgression for fitness, behaviour and interaction with other organisms are poorly understood for European wildcats. More research addressing the relationship between genomic differences and behavioural variability among cats is needed (Macdonald, Yamaguchi, et al., 2010). However, potential effects include those related to fitness reduction or genetic swamping (Todesco et al., 2016) and also may extend to wider ecological impacts (Ellington & Murray, 2015). For example, the behaviour of domesticated animals may be greatly affected by artificial selection exerted by the human lifestyle, which may not be suitable for wild animals (Driscoll & Macdonald, 2010; Driscoll et al., 2009). Moreover, hybrids may exhibit different environmental requirements and behaviours reflecting intermediate phenotypes compared to the parental taxa (Quilodrán, Montoya‐Burgos, & Currat, 2015). The effects of introgression with domesticated organisms are reinforced when the domestic group massively outnumber its wild counterparts (e.g. Hughes & Macdonald, 2013), and extinction risk soars when rare taxa hybridize with more populous ones (Quilodrán, Currat, & Montoya‐Burgos, 2014).

Conservation planning is often influenced by the legal framework in which it operates, with wildcats in Scotland providing a notable example. There, legal protection of the wildcat is contingent on its genetic and taxonomic status (Macdonald, 2019). This further pernicious consequence of hybridization may often be neglected (Leonard, Echegaray, Randi, & Vilà, 2013) but is likely to exacerbate the conservation dilemma in the Jura (Haig et al., 2011). It is therefore critical to establish the level of introgression at which genetic rescue is still possible and to define the status of hybrids under the law. However, the legal implications of wildcat and domestic cat taxonomy and the varying consequences of specific or subspecific status are in themselves already legally challenging (Macdonald, Yamaguchi, et al., 2010).

### 4.5 Conservation concerns

The European wildcat is still listed on the Red List of several countries, and the major risk is considered to be the hybridization with domestic cats (Macdonald, Yamaguchi, et al., 2010; Yamaguchi, Kitchener, Driscoll, & Nussberger, 2015). Because domestic cats exhibit phenotypic and genetic variations and a legacy of domestication related to human activities, the impact of these traits when introduced into wildcat populations is scientifically interesting and potentially relevant to conservation.

Our simulations highlight that even (and perhaps optimistically) assuming a competitive advantage of wildcats, their population size should match that of domestic cats if massive introgression is to be avoided in the next 100 years, and that, even less plausibly, the wildcat population should be twice that of domestic cats, in the area where they coexist, to avoid a massive introgression in the long term.

An increased population size of a predator may have a broad ecological impact on its interaction with other species, some of which may also be threatened (Ripple et al., 2016). The reintroduction and increasing population of wolves in Yellowstone National Park influenced a whole trophic cascade in a way that has been considered to be effective in the restoration of the ecosystem (Ripple & Beschta, 2012). While we cannot suggest a positive or negative impact of an increase in the population size of wildcats on other organisms, we can propose that conservation programmes focus on increasing the effective population size of the wildcats, which might be achieved by increasing the quantity and interconnectedness of high‐quality habitats.

An ongoing problem in discussions of wildcat conservation is the value of hybrids, which carry wild genes preserved in their partially domestic bodies (Macdonald, Daniels, Driscoll, Kitchener, & Yamaguchi, 2004; Macdonald, Yamaguchi, et al., 2010). In addition to serving as vehicles for endangered genes, hybrids may beneficially enhance some aspects of diversity (Quilodrán, Austerlitz, Currat, & Montoya‐Burgos, 2018). While our simulations project an increase in introgression, they offer no insight into the effects of such introgression on individual fitness.

Our results add force to the existing weight of information emphasizing responsible cat ownership, neutering and decreases in stray and feral cats as priorities for achieving wildcat conservation (Macdonald, Yamaguchi, et al., 2010). The Scottish Wildcat Conservation Action Plan provides a blueprint for practical action for the conservation of wildcats, including attention to neutering feral domestics, working with landowners to reduce persecution, and community engagement (Scottish Natural Heritage, 2013). Reducing the number of feral domestic cats and hybrids (e.g. through neutering programmes) is important because when these cats reach adulthood, they may easily outcompete a dispersing young wildcat, pushing it into less optimal habitats. Actions at the landscape scale allowing the habitat to support the highest possible density of wildcats may be especially efficient for wildcat conservation. Early interventions are likely to be less costly both economically—and ecologically—than later ones (Saari, Richter, Robbins, & Faeth, 2014; Todesco et al., 2016), and waiting much longer will risk irreversibility of the threat to Jura's wildcats.

## CONFLICT OF INTEREST

None declared.

## Supporting information

Appendix S1

## Data Availability

All data necessary to repeat the simulations are described in the main text and supporting information. The sequences of wildcats and domestic cats are deposited at Dryad https://doi.org/10.5061/dryad.270b7.
