# Supplementary material for: Projecting introgression from domestic cats into European wildcats in the Swiss Jura
Source: Evol Appl. 2020 May 5;13(8):2101–12. doi: 10.1111/eva.12968 (PMC7463310; doi:10.1111/eva.12968)
Supplement: Supplementary file 1 — Appendix S1 [file EVA-13-2101-s001.pdf]

Evolutionary Applications

Supporting information

Projecting introgression from domestic cats into European wildcats in the Swiss Jura

Claudio S. Quilodrán[1,2*], Béatrice Nussberger[3], David W. Macdonald[4], Juan I. Montoya-Burgos[5,6†], Mathias Currat[2,6†]

[1] Department of Zoology, University of Oxford, Oxford, United Kingdom.

[2] Laboratory of Anthropology, Genetics and Peopling History, Department of Genetics and Evolution - Anthropology Unit, University of Geneva, Geneva, Switzerland.

[3] Institute of Evolutionary Biology and Environmental Studies, University of Zurich, Zurich, Switzerland.

[4] Wildlife Conservation Research Unit, The Recanati-Kaplan Centre, Department of Zoology, University of Oxford, Oxford, United Kingdom.

[5] Laboratory of Vertebrate Evolution, Department of Genetics and Evolution, University of Geneva, Geneva, Switzerland.

[6] Institute of Genetics and Genomics in Geneva (IGE3), Switzerland.

[†] These authors contributed equally to this work.

*Corresponding author. Email: claudio.quilodran@zoo.ox.ac.uk

Appendix S1. Variable level of competition between both cats

Our simulations described in the main text to explain the current level of introgression between cats, resulted in 32,100 simulations for each genetic marker and each model of competition: with ($\alpha = 1$) or without ($\alpha = 0$) competition between both cats. We extended those simulations by including a variable value of competition (α), ranging between 0 and 1. The value of α represents a variable competitive advantage of wildcats over domestic cats because it has been proposed to be important (Gil-Sánchez et al., 2015). At contrary, the competitive advantage of domestic cats over wildcats is always set to zero ($\alpha_{domestic-wild} = 0$) because all simulations that include a competitive advantage of domestic cats over wildcats does not allow the recolonization of wildcats in the area already occupied by domestic cats (data not shown). Note that intraspecific competition is always included in our simulations.

We performed an ABC approach with a tolerance level of 5% to assess the value of α that best explains the current observed introgression (see methods). The analysis shows that various values of α (between 0 and 0.8) resulted in a similar introgression level after the number of generations simulated (Fig. S1), despite a value of α equal to 0.1 seems to increase the explanation of the introgression, when compared with a $\alpha = 0$. We thus performed a Bayesian model comparison between a model without competition ($\alpha = 0$) and a model with a competition advantage of wildcats of 10% ($\alpha = 0.1$). While a weak support is observed for the last model when considering the mtDNA and Y chromosome markers (both Bayes factor smaller than 1.5), they perform equally likely for the autosomal markers and all markers together (Bayes factor equal to or smaller than 1). In addition, both models have similar goodness of fit for all genetic markers analysed (p-values > 0.05, Table S1). This means that we are not able to discriminate between a model without competition ($\alpha = 0$) and a model with a level of competition smaller than 0.8. Values higher than 0.8 are less likely in explaining the observed introgression (Fig. S1).

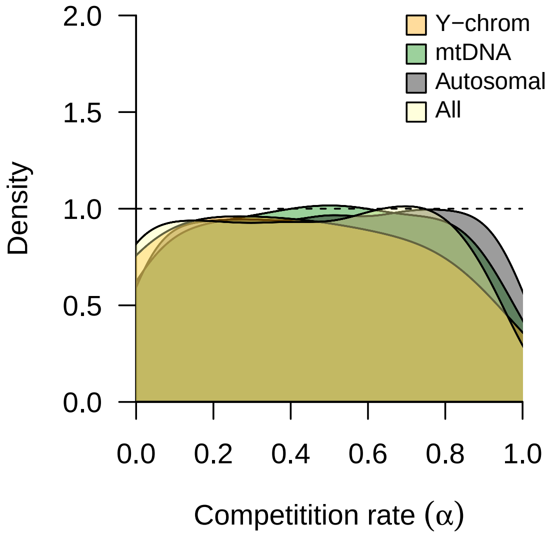

Figure S1. Bayesian estimation of the coefficient of competition (α) of wildcats over domestic cats explaining the currently observed introgression between cats.

Table S1. Approximate Bayesian computation (ABC) was used for comparison between a model without competition between cats, and a model with 10% of competition advantage of wildcats over domestic cats. Five percent of the best simulations were retained. The Bayes factor is presented as the probability of the "no competition" model (numerator) relative to the probability of each other model (denominator). The p-value of the goodness of the fit of each model (GOF) is also presented. We performed 32,100 simulations for each model of competition and genetic marker.

**Marker**	**Model**	**Posterior probability**	**Bayes factor**	**GOF p-value**
Autosomal	No competition	0.51	1	0.42
	Competition 10%	0.49	1.05	0.44
mtDNA	No competition	0.60	1	0.35
	Competition 10%	0.40	1.47	0.46
Ychrom	No competition	0.56	1	0.32
	Competition 10%	0.44	1.27	0.12
All	No competition	0.48	1	0.5
	Competition 10%	0.52	0.92	0.40

Table S2. Approximate Bayesian computation (ABC) used for model comparison at 10% and 15% of tolerance level. We performed 32,100 simulations for each model of competition and genetic marker.

		**Tolerance 10%**		**Tolerance 15%**	
**Marker**	**Model**	**Posterior probability**	**Bayes factor**	**Posterior probability**	**Bayes factor**
Autosomal	No competition	0.58	1	0.58	1
	Competition	0.42	1.35	0.42	1.37
mtDNA	No competition	1	1	0.99	1
	Competition	0	19487	0.01	128
Ychrom	No competition	1	1	0.99	1
	Competition	0	58804	0.01	980
All	No competition	0.99	1	0.9	1
	Competition	0.01	495	0.09	9.27

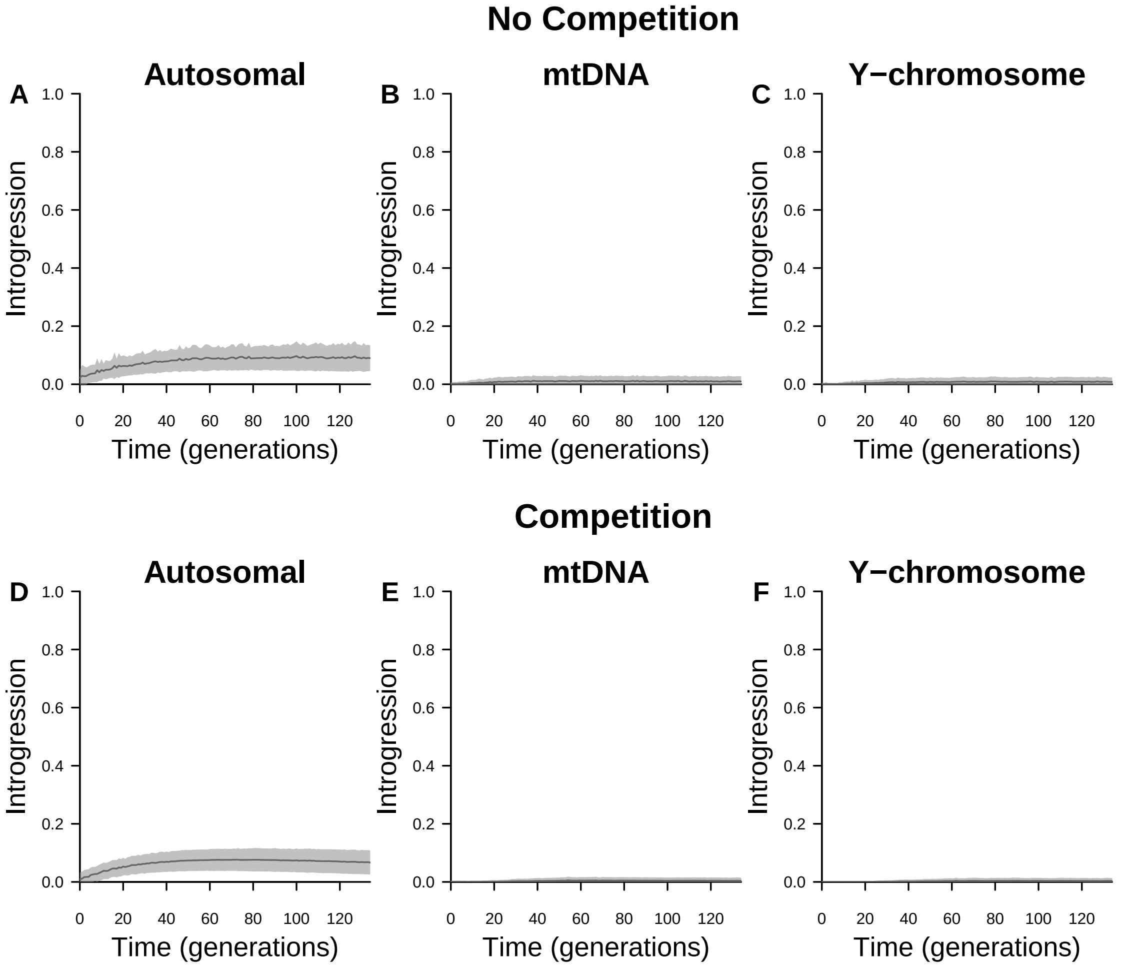

Figure S2. Projected introgression levels through time in domestic cats (mean ± SD). Top level figures project introgression in the absence of competition between cats, while bottom figures include competition between them. Competition within populations of cats is always included in the simulations (see methods). Introgression level is simulated with current conditions remaining constant (ranges, demographic parameters and hybridization rate, in a "no changes" scenario). Values are averaged over 10,000 simulations.

Literature Cited

Gil-Sánchez, J., J. Jaramillo, and J. Barea-Azcón, 2015. Strong spatial segregation between wildcats and domestic cats may explain low hybridization rates on the iberian peninsula. Zoology 118:377–385.
